# Influence of Estrogen Receptor ***α*** Polymorphisms on Bone Density in Response to Habitual Exercise in Japanese Postmenopausal Women

**DOI:** 10.1155/2014/593927

**Published:** 2014-07-23

**Authors:** Hiroyo Kondo, Hidemi Fujino, Fumiko Nagatomo, Akihiko Ishihara

**Affiliations:** ^1^Department of Food Sciences and Nutrition, Nagoya Women's University, Nagoya 467-8610, Japan; ^2^Department of Rehabilitation Science, Kobe University Graduate School of Health Sciences, 7-10-2 Tomogaoka, Kobe 654-0142, Japan; ^3^Laboratory of Cell Biology and Life Science, Graduate School of Human and Environmental Studies, Kyoto University, Kyoto 606-8501, Japan

## Abstract

Estrogen receptor *α* (*ER* *α*) is one of candidate genes for osteoporosis. This study examined the influence of *ER* *α* gene, *Pvu*II, and *Xba*I genotypes on bone density of calcaneus in response to habitual exercise. *ER* *α* polymorphisms were detected using *Pvu*II and *Xba*I restriction enzymes in 316 Japanese postmenopausal women. The bone density was significantly lower in the women carrying PP, pp, or xx genotype without habitual exercise than in the age-matched women without those genotypes. The women carrying Pp genotype without habitual exercise had normal bone density compared to those without Pp genotype. The women carrying PPxx or ppxx polymorphism without habitual exercise had low bone density compared to those with habitual exercise. Thus, the reduction of bone density was attenuated in the women carrying PPxx or ppxx with habitual exercise. In addition, habitual exercise was highly effective for the bone density in the women carrying xx homozygote. These findings indicate that analyses of *Xba*I and *Pvu*II polymorphisms of *ER* *α* may be useful to predict the effect of exercise on bone density, and habitual exercise attenuates the reduction of bone density in women with some genotypes.

## 1. Introduction

Osteoporosis is one of skeletal disorders that predispose to increased risk of fractures and is a multifactor disease caused by lifestyle and multiple genetic factors. The lifetime risk of osteoporotic fractures is estimated to be 40–50% for elderly women [1]. Not only environmental and hormonal but also genetic factors have effects on bone density; for example, family and twin studies [2, 3] have shown that genetic factors may explain 70% of bone mineral density variability. Regarding the role of genetic predispositions in osteoporosis, the vitamin D receptor polymorphism concerning the bone density in postmenopausal women was reported [4]. In addition, a follow-up study [5] has been performed to evaluate whether bone density is affected by vitamin D receptor because bone homeostasis is known to be regulated by the vitamin D secretion system. The role of vitamin D receptor polymorphism in the bone density was reported to be 2.5% in a meta-analysis [6]. Genes that appear to be important for bone homeostasis include calcium-regulating hormone receptors such as estrogen receptor *α* (*ER* *α*), cytokines [7, 8], and other bone-related proteins [9]. Estrogen has a direct action on osteoblast-osteoclast and an indirect action on 1*α*25-dihydroxyvitamin D_3_ (1,25(OH)_2_D_3_) and parathyroid hormone [10]. The osteoprotective actions of estrogens are clearly demonstrated by postmenopausal osteoporosis [11] and *ER* *α* null mouse model [12]. In addition, *ER* *α* null mice could not increase bone mass in response to mechanical loading [12]. Thus, *ER* *α* could play important roles in the maintenance of bone metabolism. Moreover, several studies [13–15] have addressed the relationship between* Xba*I and* Pvu*II polymorphisms of *ER* *α* gene and bone density in pre- and postmenopausal women. The association between *ER* *α* gene and bone density was reported [16]; however, Han et al. [15] found no relation. These reports indicate that bone mass and bone loss are determined by a complex interaction between genetic and environmental factors, such as habitual exercise.

It has been widely established that physical activity and/or exercise is an important regulator maintaining bone mass. Previous studies [17, 18] indicate that exercise can improve bone density and decrease the number of fractures in elderly persons. These studies indicate that the difference in the bone density among individuals may be influenced by environmental factors such as physical activity or the characteristics of individuals. In addition, several studies [19, 20] focused on the relationship between exercise and gene polymorphisms and indicated that exercise is associated with bone density and vitamin D receptor polymorphism. Furthermore, the adaptive responses of bone to mechanical stress require functional *ER* *α* [12], and the *ER* *α* genotypes are associated with an increase in the bone density [21], whereas there is no relationship between bone density and *ER* *α* polymorphisms in young healthy men [22]. The controversy might be explained, at least in part, by genetic factors as well as their interactions with the level of physical activities. Therefore, this study examined the effect of habitual exercise on the bone density in Japanese postmenopausal women with *ER* *α* gene polymorphisms.

## 2. Materials and Methods

### 2.1. Subjects

Healthy postmenopausal women aged 50–80 years (*n* = 316, 61.2 ± 0.4 years) living at the region of central Japan were enrolled in this study. None of the women had the history of metabolic bone disease or previous use of pharmaceutical agents that could affect bone turnover. All women were asked to report the average amount of time spent per week during the previous years in the habitual exercise, for example, walking, hiking, jogging, running, bicycling, racquet sports, swimming, or other activities. Each exercise in the questionnaire was assigned a metabolic equivalent (MET) score based on the classification by Anisworth et al. [23]. All women were categorized into two groups according to habitual exercise history: (1) exercise group that performed habitual exercise (≥3 METs × 4 h/wk) for at least 1 year and (2) nonexercise group that did not perform habitual exercise for at least 1 year.

There were no significant differences in physical characteristics between these 2 groups (Table 1). This study was approved by the Institutional Human Ethics Committee of Suzuka University of Medical Science and Nagoya Women's University.

### 2.2. Analysis of *ER* *α* Genotypes

Buccal mucosal cells were harvested from subjects after obtaining informed consent. Buccal mucosal cells were collected from the subjects with 20 strokes using cytology brush (CytoSoft, Medical Packaging, Camarillo, CA) and genomic DNA was extracted using Gentra Puregene Buccal Cell Kit (Qiagen Valencia, CA). The *ER* *α* genotype was detected by restriction fragment length polymorphism (RFLP) using endonucleases (*Pvu*II and* Xba*I) and polymerase chain reaction (PCR) primers as described by Becherini et al. [24].* Pvu*II and* Xba*I recognize the restriction sites CAGCT (allele C/T) and TCTAGA (allele A/G), respectively. To assess polymorphisms in intron 1 of the *ER* *α* gene of human chromosome 6 (6 q 25.1), 100 ng of DNA was amplified using 50 *μ*L of buffer solution and 1 U of Taq polymerase (Promega, Madison, WI). The oligonucleotide primers used in this study were as follows:

 forward primer: 5′-CTGCCACCCTATCTGTCTTTTCCTATTCTCC-3′,
 reverse primer: 5′-TCAGATAATCGACGCCAGGGTGGCAGAGAAAGA-3′.

The protocol was followed by Kobayashi et al. [16]. Thirty cycles of PCR were performed with the following steps: denaturation at 94°C for 30 sec, annealing at 60°C for 1 min, and extension at 72°C for 90 sec. The product included parts of intron 1 and exon 2 of the ER gene. After amplification, the product was digested at 37°C for 2 h with 10 U of either* Pvu*II or* Xba*I restriction endonucleases (Toyobo, Osaka, Japan) and then electrophoresis was performed in a 1.0% agarose gel. Gene analyses were carried out to identify the genotype. Lower case letters (p and x for* Pvu*II and* Xba*I endonucleases, resp.) were used to indicate the presence of restriction site for each endonuclease, and upper case letters (P and X) were used to indicate the absence of restriction sites. Subjects were categorized as pp or xx homozygote, Pp or Xx heterozygote, and PP or XX homozygote according to the digestion pattern. In addition, classification by haplotype combination was performed.

### 2.3. Measurement of Bone Density

Quantitative ultrasound (QUS) measurement using AOS-100 (Aloka, Tokyo, Japan) was performed for evaluation of the bone density of the right calcaneus. Three ultrasonic parameters were measured: (1) the speed of sound (SOS), (2) the transmission index (TI), and (3) the osteosonoassessment index (OSI). TI was reported as QUS (UBA575, Walker Sonix, Naples, FL), which was shown to have a good correlation between TI and bone mineral density [25]. Moreover, QUS instruments have advantages compared with dual-energy X-ray absorptiometry (DXA); they are radiation-free, portable, and inexpensive [26]. QUS bone measurement has been recognized by the American Food and Drug Administration (FDA) and has become the world standard [27]. The OSI was calculated by SOS and TI reflecting bone density and structure. The *Z* score (SD) used for the statistical analysis represents the proportion of the standard values for each age of OSI, and the variability by age was corrected by this method. Accuracy of the data was in the range of 1% based on values obtained by 3 measurements.

### 2.4. Statistical Analysis

Data were expressed as the mean ± SEM. The difference between two groups was evaluated by unpaired two-tailed Student's *t*-test. Differences between *ER* *α* genotype and bone density (*Z* score) were evaluated by ANOVA and post hoc analysis using Kruskal-Wallis with Mann-Whitney *U* test (Bonferroni correction). The effect of exercise on the same polymorphism was calculated by Mann-Whitney *U* tests. The *P* values of <0.05 were considered statistically significant. All analyses were performed using the statistical software package SPSS for Windows (Version 15.0 SPSS, Chicago, IL).

## 3. Results

### 3.1. Physical Characteristics and Bone Density (Z Score) in the Exercise and Nonexercise Groups

**Figure 1:**
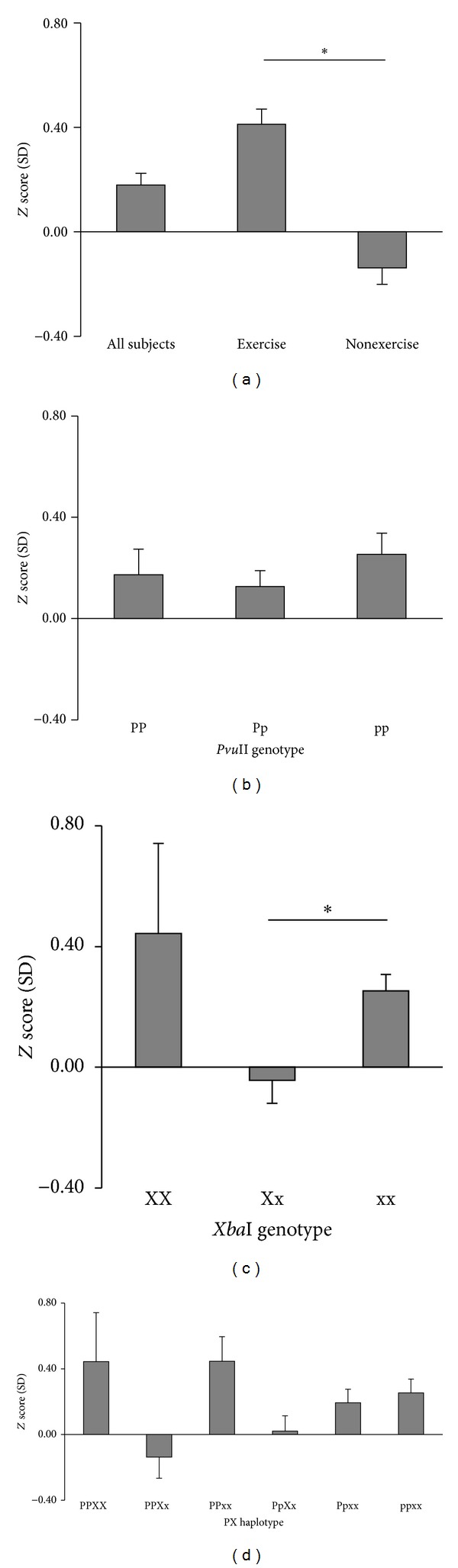
Dependence of bone density *Z* scores on* Pvu*II and* Xba*I RFLP genotypes and haplotypes. Values are expressed as mean ± SEM. The zero line is equal to the age-based mean value. *P < 0.05 compared between the exercise and nonexercise groups in each *ER* *α* genotype and haplotype. (a) The *Z* score (SD) of the exercise group was significantly higher than that of the nonexercise group. (b) pp showed higher *Z* score than Pp, but there was no significant difference. (c) In the* Xba*I genotype, the *Z* score of xx was significantly higher than that of Xx genotype. (d) The *Z* score of PPXX and Ppxx was significantly higher than other genotypes, but there was no significant difference.

The physical characteristics of the women are shown in Table 1. There were no significant differences in age, height, body weight, or body mass index (BMI) between the exercise and nonexercise groups (Table 1). The *Z* score of the exercise group was significantly higher than that of the nonexercise group, indicating that habitual exercise attenuates the reduction of bone density in the calcaneus (Figure 1(a)).

### 3.2. Allelic Associations of* Pvu*II*/Xba*I RFLPs in *ER* *α*

The genotype distributions in cases and controls followed the Hardy-Weinberg equilibrium, indicating that subjects had homogenous genetic backgrounds. In the* Pvu*II RFLP analysis (632 chromosomes), there were 77 women with PP homozygote (21%), 101 women with pp homozygote (29%), and 138 women with Pp heterozygote (50%). The RFLP analysis of* XbaI* determined 15 women with XX homozygote (4%), 212 women with xx homozygote (65%), and 89 women with Xx heterozygote (31%). The haplotype analysis of* Pvu*II and* Xba*I genotypes disclosed the following results: PPXX (4.7%) in 15 women, PPXx (11.4%) in 36 women, PPxx (8.2%) in 26 women, PpXx (16.8%) in 53 women, Ppxx (26.9%) in 85 women, and ppxx (32.0%) in 101 women. The haplotypes of ppXx, ppXX, and PpXX were not detected in this study.

### 3.3. Influence of *ER* *α* Genotype on the Bone Density

The *Z* score was increased in the order of pp > PP > Pp in the* Pvu*II genotype (Figure 1(b)). The *Z* score of bone density in pp or PP genotype was higher than that in Pp genotype although there was no significant difference among groups. In the* Xba*I genotype, the *Z* score of bone density in xx genotype was significantly higher than that in Xx genotype (Figure 1(c)). The *Z* score in PPxx and PPXX haplotypes was higher than that in other haplotypes, whereas that of PPXx was lower than the age-based mean value (Figure 1(d)). In particular, the difference in mean *Z* score value between the PPXx and PPxx genotypes was 0.58 although there was no significant difference with Bonferroni correction.

### 3.4. Effect of Habitual Exercise on the Bone Density

**Figure 2:**
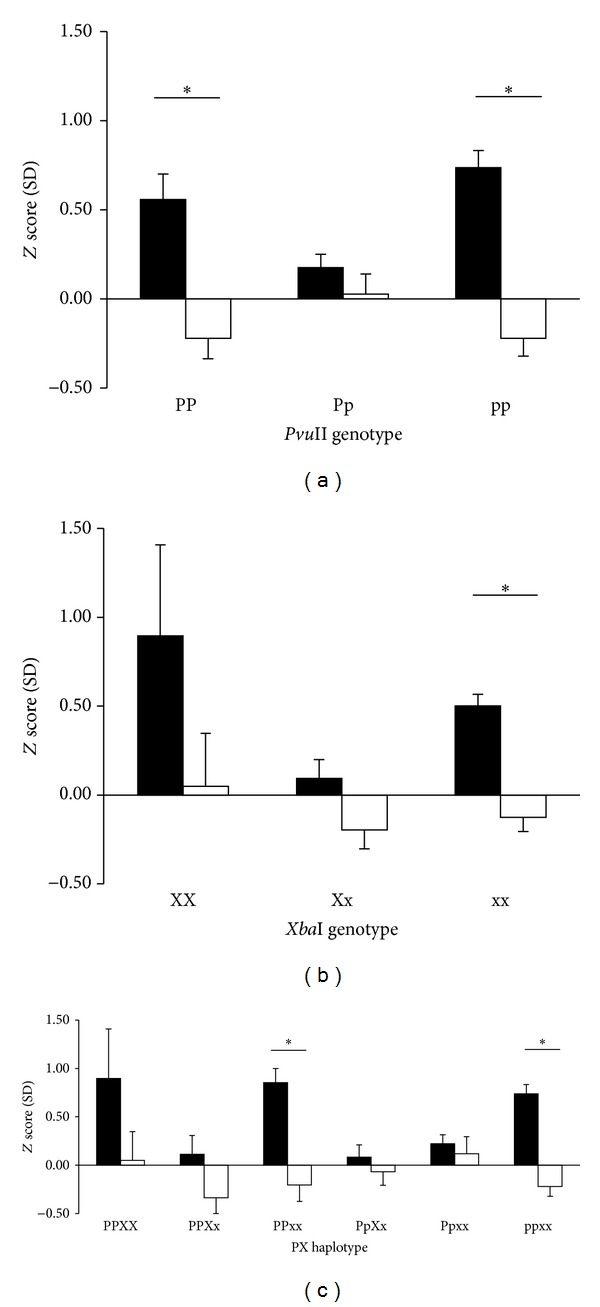
The exercise effect on bone density *Z* scores classified by* Pvu*II and* Xba*I RFLP genotypes and haplotypes. Values are expressed as mean ± SEM. The zero line is equal to the age-based mean value. Solid and open bars indicate the exercise and nonexercise groups, respectively. *P < 0.05 compared between the exercise and nonexercise groups in each *ER* *α* genotype and haplotype. (a) The mean *Z* score in the PP or pp genotype with exercise was significantly higher than that without exercise. (b) The* Z* score in the exercise group with xx genotypes was significantly higher than that of the nonexercise group. (c) The *Z* score in the exercise group with PPxx or ppxx genotype was significantly higher than that of the nonexercise group. The difference of habitual exercise in bone density was minimal in PpXx and Ppxx.

Bone density was evaluated by each *ER* *α* genotype and compared between the exercise and nonexercise groups (Figure 2). In the* Pvu*II polymorphism, the *Z* score of bone density in the exercise group in the women carrying either pp or PP genotype was significantly higher than that in the nonexercise group in the women carrying the same genotype, whereas there was not a significant difference in the *Z* score of bone density between the exercise and nonexercise groups in the women carrying Pp genotype (Figure 2(a)).

In the* XbaI* polymorphism, the *Z* score of bone density in the exercise group in the women carrying xx genotype was significantly higher than that in the nonexercise group in the women carrying the same genotype, whereas there was not a significant difference in the *Z* score of bone density between the exercise and nonexercise groups in the women carrying either XX or Xx genotype (Figure 2(b)).

In the* Pvu*II and* Xba*I haplotypes, the *Z* score of bone density in the exercise group in the women carrying either ppxx or PPxx haplotype was significantly higher than that in the nonexercise group in the women carrying the same haplotype (Figure 2(c)). In addition, the *Z* score in the nonexercise groups carrying PPXx, PPxx, and ppxx genotypes was lower than that of the age-based mean value.

## 4. Discussion

In this study, we found that the bone density of heel calcaneus was improved by habitual exercise, and this improvement depends on the presence of* Pvu*II and* Xba*I genotypes. A possible benefit of habitual exercise was observed only in women carrying the PPxx or ppxx haplotype, whereas no strong benefit was observed in women with other haplotypes. Thus,* Pvu*II and* Xba*I analysis predicted whether habitual exercise would be effective for increasing and/or maintaining the bone density in postmenopausal women. Our present study is the first to find differences in bone density in women carrying* Pvu*II and* Xba*I genotypes and PX haplotype in response to habitual exercise. In particular, the haplotype with xx homozygosity had a decrease in bone density without habitual exercise, whereas the bone density was not decreased to values less than the age-based mean value in the haplotypes with Pp heterozygosity. Interestingly, we found that exercise exerts no similar effects on bone density for all women in the present study, but it depends on the type of *ER* *α* genotypes.

It is well known that mechanical stimulation from habitual exercise modulates bone remodeling [28] and minimizes postmenopausal bone loss [29]. In this study, we demonstrate that postmenopausal women that performed habitual exercise have high bone density compared to the women in the nonexercise group. Thus, habitual exercise is associated with higher bone density. In addition, the Nurses' Health Study of postmenopausal women suggested that the relative risk of fracture was reduced by 6% for every 3 METs × 4 h/wk of physical activity, which is roughly equivalent to 1 h of walking per week [30]. This report indicates that habitual exercise trials effectively reduced risk of fracture in postmenopausal women. Therefore, habitual exercise may be most effective in developing and maintaining skeletal integrity and minimizing fracture risk [31].

For the* Xba*I and* Pvu*II polymorphisms, the XX genotype conferred a highly significant protection against the overall fracture risk in elderly European women [32], whereas Suuriniemi et al. [33] reported that the pubertal Finnish girls carrying the Pp genotype with strong physical activity have high bone density, while the bone density is not affected by physical activity in the girls with PP and pp homozygotes. However, our present results suggested that the women carrying PP and pp genotypes that performed habitual exercise have high bone density compared to the women in the nonexercise group. Thus, Japanese women carrying PP and pp homozygotes were highly responsive to physical activity compared to the women carrying Pp genotypes. These observations may suggest that habitual exercise has effects on bone formation in pubertal women with* Pvu*II heterozygote and the habitual exercise has an effect on the prevention of bone loss in elderly women with homozygote.

The habitual exercise was not of benefit to the bone density of women carrying the PpXx, Ppxx, or PPXx haplotype, whereas the habitual exercise was of strong benefit to the bone density of women with the ppxx or PPxx haplotype. The bone density in women with the ppxx or PPxx haplotype was higher than the age-based mean value in the exercise group, whereas that was lower than the age-based mean value in the nonexercise group. Our present data are consistent with the findings reported by several studies [8, 34]. Yamada et al. [8] reported the relationship between TC (*Pvu*II) and AG (*Xba*I) polymorphisms and bone mineral density in Japanese women. In women (≤ 60 years old), the bone density of femoral neck was significantly lower in the postmenopausal women carrying CC/GG (ppxx) genotype than in those carrying other genotypes [8]. Thus, these findings suggest that the postmenopausal women carrying those genotypes, except PPXx, can improve their bone density by habitual exercise. On the contrary, the *Z* scores in the exercise women carrying PPXx, PPxx, PpXx, and ppxx genotypes were lower than the age-based mean value in the present study. This finding suggests that the postmenopausal women carrying those genotypes cannot improve their bone density by habitual exercise. Particularly, it is worthy to note that the *Z* score in the nonexercise women carrying PPXx genotype was the lowest (−0.34 SD). This observation suggests that the nonexercise women carrying PPXx have a risk of developing osteoporosis.

Exercise is known to enhance the bone density of heel because gravity force loads the heels. Indeed, the habitual exercise showed a significant increase in the bone density of heel in our present cross-sectional study. However, the exercise that increases bone density may not be always of high impact-loading type, and various types of exercise may increase bone density because a low impact-loading type, for example, swimming, was also included in the present study. In addition, the *ER* *α* protein is expressed in a variety of cell types including human bone cells [35].

In conclusion, the present study showed that* Pvu*II and* Xba*I polymorphisms of the estrogen receptor gene affect differently the maintenance and development of bone mass in response to exercise. The haplotype with PP, pp, and xx homozygotes was found to improve bone mass by habitual exercise, and the haplotype with Pp and Xx heterozygotes was found to prevent the decrease of bone mass to less than the age-based mean value. These results suggest that *ER* *α*genotype may influence the relationship between habitual exercise and bone density.

## Figures and Tables

**Table 1:** Physical characteristics in the exercise and nonexercise groups.

	All subjects (*n* = 316)	Exercise (*n* = 182)	Nonexercise (*n* = 134)
Age, yr	61.24 ± 6.96	60.55 ± 5.92	62.18 ± 8.11
Height, m	1.54 ± 0.06	1.54 ± 0.06	1.54 ± 0.06
Body weight, kg	54.05 ± 7.28	54.55 ± 6.66	53.34 ± 8.06
BMI	22.80 ± 3.22	22.96 ± 3.07	22.60 ± 3.40

Values are means ± SD. BMI: body mass index (body weight, kg/height squared, m^2^).
